# In Vitro, In Vivo, Ex Vivo Characterisation of Dihydroimidazotriazinones and Their Thermal Decomposition Course Studied by Coupled and Simultaneous Thermal Analysis Methods

**DOI:** 10.3390/ijms26020541

**Published:** 2025-01-10

**Authors:** Marta Worzakowska, Małgorzata Sztanke, Jolanta Rzymowska, Krzysztof Sztanke

**Affiliations:** 1 Department of Polymer Chemistry, Institute of Chemical Sciences, Faculty of Chemistry, Maria Curie-Skłodowska University in Lublin, 33 Gliniana Street, 20-614 Lublin, Poland; marta.worzakowska@mail.umcs.pl; 2 Department of Medical Chemistry, Medical University of Lublin, 4A Chodźki Street, 20-093 Lublin, Poland; 3 Department of Biology and Genetics, Medical University of Lublin, 4A Chodźki Street, 20-093 Lublin, Poland; jolanta.rzymowska@umlub.pl; 4 Laboratory of Bioorganic Compounds Synthesis and Analysis, Medical University of Lublin, 4A Chodźki Street, 20-093 Lublin, Poland; krzysztof.sztanke@umlub.pl

**Keywords:** dihydroimidazotriazinones, anticancer activity, in vivo, in vitro and ex vivo toxicity profile, DSC/TG/DTG/FTIR/QMS, decomposition course, thermal behaviour

## Abstract

The biological and thermal properties of a class of synthetic dihydroimidazotriazinones were disclosed in this article for the first time. Molecules **1**–**6**—as potential innovative antimetabolites mimicking bicyclic aza-analogues of isocytosine—were evaluated for their in vitro anticancer activity. Moreover, in vivo, in vitro, and ex vivo toxicity profiles of all the compounds were established in zebrafish, non-tumour cell, and erythrocyte models, respectively. Their antihaemolytic activity was also evaluated. Additionally, the thermal decomposition mechanism, path, and key thermal properties of heterocycles **1**–**6** were analysed. It was found that all the studied compounds revealed significant antiproliferative activities against tumour cells of the lung, cervix, ovary, and breast, as well as acute promyelocytic leukaemia cells, superior or comparable to that of an anticancer agent gemcitabine. Most of them were less toxic to non-tumour cells than this standard drug, and none had a haemolytic effect on red blood cells. All the tested heterocycles proved to be safer for zebrafish than a standard drug pemetrexed. Some exhibited the ability to inhibit oxidative haemolysis, suggesting their protective action on erythrocytes. The differential scanning calorimetry (DSC) analyses confirmed that all molecules melted within one narrow temperature range, proving their purity. The melting points depended solely on the type of substituent and increased as follows: **4** (R = 3-ClPh) < **2** (R = 4-CH_3_Ph) = **3** (R = 4-OCH_3_Ph) < **5** (R = 4-ClPh) = **1** (R = Ph) < **6** (R = 3,4-Cl_2_Ph). The thermogravimetry/differential thermogravimetry (TG/DTG) studies confirmed high thermal stability of all the investigated heterocycles in inert (>230 °C) and oxidising (>260 °C) atmospheres, which depended directly on the R. The pyrolysis process included one main decomposition stage and was connected with the emission of NH_3_, HCN, CH_3_CN, HNCO, alkane, alkene, aromatic fragments, CO_2_ (for all the compounds), and HCl (for the molecule with 3,4-Cl_2_Ph), which was confirmed by FTIR and QMS analyses. In turn, the oxidative decomposition process of the tested polyazaheterocycles took place in two main stages connected with the formation of the same volatiles as those observed in an inert atmosphere and additionally with the release of N_2_, NO, CO, and H_2_O. These results proved that the pyrolysis and oxidative decomposition run through the radical mechanism connected with the additional reactions between radicals and oxygen in synthetic air. The favourable biological and thermal properties of this class of dihydroimidazotriazinones imply their usefulness as potential pharmaceutics.

## 1. Introduction

**Figure 1:**
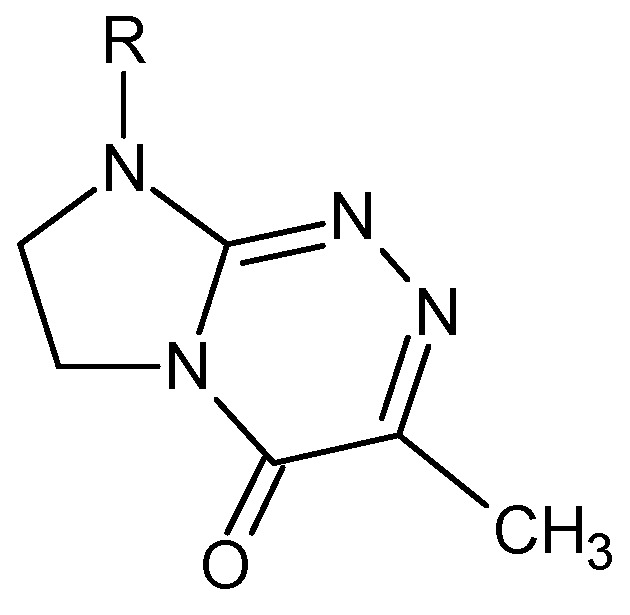
Structures of the investigated compounds: **1**. R = Ph; **2.** R = 4-CH_3_Ph; **3**. R = 4-OCH_3_Ph; **4**. R = 3-ClPh; **5.** R = 4-ClPh; **6**. R = 3,4-Cl_2_Ph.

3-Methyl-8-(R-phenyl)-7,8-dihydroimidazo[2,1-*c*][1,2,4]triazin-4(6*H*)-ones (**1**–**6**) (Figure 1), the structure of which has been established with the use of ^1^H NMR and EI-MS spectra [1], mimic polyazaheterocyclic nucleobases of the antimetabolite-type. They may be incorporated as false building blocks during the DNA synthesis phase, thereby inhibiting the action of enzymes involved in biochemical pathways and leading to lethal synthesis, or they may form pairs with purine and pyrimidine nucleobases, thus acting as transcription inhibitors. Due to the locked-up amine nitrogen in the ring, their molecules may be resistant to metabolic deamination, unlike some clinically approved antimetabolites that are short-acting because they contain a free amino group outside the ring, susceptible to deaminases. These innovative molecules may become of interest to biologists and pharmacologists, as some of the antimetabolites approved for clinical use are helpful in treating human malignancies such as leukaemia and solid tumours [2].

Based on the above structural assumptions and literature data [2,3], conducting biological studies—which are the novelty of the current paper—on this class of molecules, which may be potential innovative antimetabolites is fully justified and necessary. Both the pharmacological activity and toxicity profile of dihydroimidazotriazinones **1**–**6** have not been studied and described so far. Therefore, the present biological investigation is aimed at determining the antiproliferative action of nucleobase-mimicking molecules against human solid tumour cells of the lung, cervix, ovary, and breast, as well as acute promyelocytic leukaemia cells. Additionally, it is advisable to examine whether dihydroimidazotriazinones are able to counteract haemolysis induced by some reactive oxygen species. The rationale for anticancer and antihaemolytic investigations is the fact that structurally related annelated triazinones described earlier exhibited a broad spectrum of anticancer activity in human tumour cells and were capable of inhibiting the oxidative haemolysis [3]. To further develop new chemical entities with promising biological activity as potential drugs, they must be thoroughly tested—in the preclinical phase—for their safety and toxicity. Therefore, it is necessary to conduct multidirectional toxicological studies. Preclinical assessment of the toxicity/safety profile of compounds with potential therapeutic use requires testing on various—animal as well as cellular—experimental models. Based on this, the goal of the research is also to assess the toxicity of each compound to animals and cells. The effect of the title molecules on mortality, hatchability, heart rate, and developmental abnormalities in the early-life stages of zebrafish (*Danio rerio*) will be assessed. Additionally, their influence on erythrocytes and non-cancerous cells will be investigated. This should enable the choice of the most selective molecules, which will be suitable for further, more extended in vivo studies. Moreover, in silico methods are helpful in assessing the toxicity of pharmacologically active compounds. These tools allow for the identification of fragments in the structure that determine a specific toxic effect (toxicophores), and they warn about so-called structural alerts. The use of computer software during safety studies of potential drugs in preclinical phase enables the indicating areas that require precise in vivo assessment. This makes the selected molecules even safer and more likely to be used in therapy. Therefore, the next purpose of the investigations is to predict the possibility of adverse effects in the studied class of dihydroimidazotriazinones.

A very important parameter when assessing the therapeutic usefulness of potential drugs is their thermal stability. Thermal characterisation of pharmaceutically active substances and excipients is required to establish their recommended dosage forms [4]. In turn, knowledge about the detailed thermal properties of potential pharmaceuticals in the preclinical phase of drug development is essential for their further improvement. Previously, some thermal studies have been carried out on structurally related isopropylated-fused triazinones to explain their thermolysis mode, thermostability, and thermal properties [5]. However, there is a research gap in the current state of knowledge about the title compounds because their thermal behaviour is unknown and no molecule from this class has been thermally studied to date. Therefore, the novelty of this part of the present paper is a detailed thermal characterisation of this set of dihydroimidazotriazinones, aimed at explaining their thermal decomposition mechanism and path. Coupled and simultaneous thermal techniques will be applied with the goal of determining the differential scanning calorimetry (DSC) curves, melting enthalpy values, and thermal stability ranges, as well as identifying the volatile degradants released under pyrolysis in inert and oxidising conditions. These multi-component methods enable the purity control of the studied compounds and verification whether their solid-state molecules do not undergo polymorphic transformations. The rationale for using simultaneous thermal techniques coupled with analytical methods is the fact that they allow the reliable interpretation of every phenomenon in the heated sample occurring with mass loss and energy changes. Besides, thermal analysis techniques, such as the simultaneous thermal analysis with the evolved gas analysis, are recommended not only for thermal studies of active pharmaceutical ingredients and their dosage forms but also are essential in thermal testing of potential drug candidates in the preclinical phase of drug development [4,6,7]. Understanding the thermal decomposition mechanism and path of this class of dihydroimidazotriazinones may be a significant contribution to the current state of knowledge. In addition, the results obtained in this study may have practical usefulness. Primarily, they will help determine the optimal storage conditions for a whole class of compounds studied. Secondly, the documented degradation profiles of these polyazaheterocycles will be crucial to establish the optimal thermal incineration conditions for their safe disposal without any environmental impact.

## 2. Results and Discussion

### 2.1. Antitumour Activity and Cytotoxicity to Non-Tumour Cells of the Tested Compounds (***1**–**6***)

The investigated class of nitrogen-bridgehead analogues of nucleobases (with similarity to potential innovative antimetabolites) includes the parent structure **1** and its five derivatives (**2**–**6**). The in vitro antiproliferative activity of all heterocycles was assessed against five cancerous cell lines: four derived from human solid tumours of the lung (A549), cervix (HeLa), ovary (TOV 112D), and breast (T47D) and one derived from human acute promyelocytic leukaemia (HL-60). In turn, the cytotoxicity of molecules to non-tumour cells was evaluated using an African green monkey kidney cell line (GMK). Antitumor activities and cytotoxicities to normal cells of dihydroimidazotriazinones **1**–**6**, as well as gemcitabine (an anticancer agent belonging to the cytidine analogues), are provided in Table 1.

The vast majority of dihydroimidazotriazinones revealed a significant growth inhibition in A549, HeLa, TOV112D, T47D, and HL-60 cell lines, suggesting that an unsubstituted phenyl ring, as well as this moiety containing electron-donating (i.e., 4-CH_3_ or 4-OCH_3_) or electron-withdrawing (i.e., 3-Cl, 4-Cl, or 3,4-Cl_2_) groups, are necessary to ensure the effectiveness against these types of tumour cells. Most molecules proved to be more antiproliferative active against A549, HeLa, TOV112D, and T47D cells than against HL-60 cells. However, HeLa and TOV112D cell lines were the most susceptible to all the investigated compounds.

Molecule **6** (bearing the 3,4-Cl_2_Ph) proved to be the most active against A549 cells, and its activity was comparable to that of gemcitabine. Compounds **5** (bearing the 4-ClPh), **3** (containing the 4-OCH_3_Ph), **4** (with the 3-ClPh), and **6** revealed higher antiproliferative activity against HeLa cells than that of gemcitabine. In addition, the parent structure (**1**) and its 4-CH_3_Ph derivative (**2**) showed twofold greater growth inhibition after 24 h of incubation than that standard drug. All dihydroimidazotriazinones revealed a significant antiproliferative effect on TOV112D and T47D cells, which was superior or comparable to that of gemcitabine. Compound **6** revealed the highest growth inhibition in HL-60 cells among all the molecules that were tested, and its antileukaemic activity was better than that of a standard drug.

Comparing the toxicity to non-tumour GMK cells of derivatives **2**–**6** in relation to parent compound **1**, it was shown that the unsubstituted phenyl moiety in **1** and the 3,4-dichloro-substituted phenyl ring in **6** are favourable in terms of selectivity. Both heterocycles (**1** and **6**) revealed a significant decrease in cytotoxicity after 48 and 72 h of incubation in relation to gemcitabine. Additionally, molecules **3**–**5** after 48- and 72-h incubation periods proved to be slightly less toxic to non-tumour cells than a standard drug. In turn, compound **2** revealed an increase in cytotoxicity only after 72 h of incubation.

Concluding, these in vitro antitumour screening data, as the first-line evidence, indicate that all the investigated innovative bicyclic azanucleobases related to isocytosine may have potential in the treatment of some human solid tumours, as well as in the chemotherapy of human acute promyelocytic leukaemia. This is consistent with the fact that some antimetabolites approved for clinical use are helpful in the treatment of human malignancies such as leukaemia and solid tumours [2,8,9]. Furthermore, five dihydroimidazotriazinones (**1**, **3**–**6**) proved to be less toxic to non-tumour cells than an anticancer agent gemcitabine; therefore, these potential antimetabolite-type molecules may be subjected to further, more extended in vivo investigations.

### 2.2. Assessment of the In Vivo Toxicity Profile of the Investigated Compounds (***1**–**6***)

Taking into account the anticancer activity and selectivity of innovative polyazanucleobases (**1**–**6**), their toxicity profile in various—in vivo animal (zebrafish—*Danio rerio*) and ex vivo cellular (red blood cells)—models was also determined.

The *Danio rerio* is commonly used in studies assessing the toxicity of chemical substances in the preclinical phase of drug development. The zebrafish embryo is considered an important model for vertebrate development. Its body is transparent, which facilitates the observation of organ formation, as well as detection of abnormalities and developmental delays. In addition, the cellular and molecular processes of zebrafish embryo development are similar to those in humans. Therefore, the effects of potential drugs on embryo development and their survival are being investigated [10,11,12,13,14,15].

To assess the toxicity/safety profile of the title molecules in the zebrafish model, some lethal (mortality) and sublethal (hatchability, heart rate, and developmental abnormalities) endpoints were examined.

**Figure 2:**
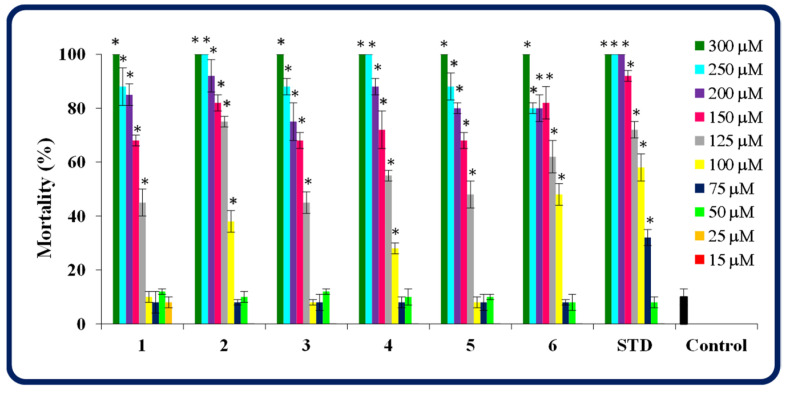
Zebrafish mortality in the control and compound/standard drug-treated groups at the end of the exposure period. STD—a standard drug pemetrexed. Data represent the mean ± SD of three independent experiments under similar conditions. *—statistically different from the control group (*p* < 0.05, Student’s *t*-test).

According to OECD guidelines [16], the coagulation of embryos, no somite formation, no detachment of the tail bud from the yolk sac, and no heartbeat are lethality indicators. In our study, the death of zebrafish was confirmed by observing (under a microscope) the absence of a heartbeat for 1 min. The effect of dihydroimidazotriazinones **1**–**6** and a standard drug pemetrexed at various concentrations on zebrafish mortality at the end of the exposure period (96 h post-fertilisation) was concentration-dependent, as shown in Figure 2. It was demonstrated that compounds **1**, **3,** and **5** at concentrations up to 100 µM and **2**, **4,** and **6**—up to 75 µM—did not significantly induce the mortality of zebrafish compared to the control group. Higher concentrations of molecules significantly reduced survival rates in zebrafish, while 100% mortality was only observed at the highest concentrations tested: 300 µM (in the case of all compounds) and 250 µM (in the case of molecules **2** and **4**). It is worth noting that the standard drug did not exhibit significant lethal effects at concentrations only up to 50 µM, and the total mortality was observed even at 200 µM. Considering the mortality rates in compound/pemetrexed-treated groups, the maximal non-lethal concentration (MNLC) and the half-maximal lethal concentration (LC_50_) were calculated for each tested compound and standard drug (Table 2). The MNLC values for molecules **1**–**6** ranged from 75 to 117 µM and were the highest for **3** and lowest for **2**, whereas the LC_50_ values were found to be from 115 (**2**) to 153 µM (**3**). Both the MNLC and LC_50_ values for pemetrexed were lower (58 and 104 µM, respectively), indicating that dihydroimidazotriazinones **1**–**6** are less toxic and therefore safer for zebrafish than this standard drug.

**Figure 3:**
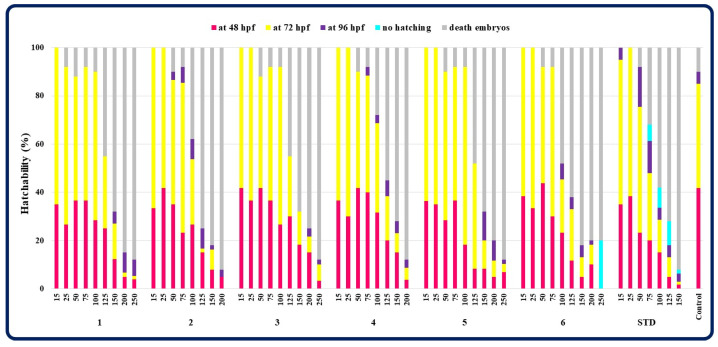
Hatching rates of zebrafish embryos in the control and compound/standard drug-treated groups. STD—a standard drug pemetrexed. hpf—hours post-fertilisation.

Hatching is precisely regulated by miscellaneous factors and is very sensitive to numerous chemicals. Exposure of zebrafish to various substances may result in accelerated, delayed, or inhibited hatching. Therefore, this parameter is used to assess the acute toxicity of chemical compounds, including potential drugs [10,11,14]. Our study showed that the exposure of zebrafish to dihydroimidazotriazinones **1**–**6** had no adverse effect on their ability to hatch (Figure 3). At 72 hpf, the majority of viable zebrafish from the untreated and compound-treated groups hatched. Interestingly, at the end of the exposure period (96 hpf), all viable embryos exposed to molecules **1**, **3,** and **5**, even at concentrations as high as 250 µM as well as those exposed to heterocycles **2**, **4**, and **6** at concentrations up to 200 µM, left the chorion. However, the hatchability of embryos exposed to structure **6** at 250 µM was inhibited, similar to the zebrafish groups treated with pemetrexed at concentrations ≥75 µM.

**Figure 4:**
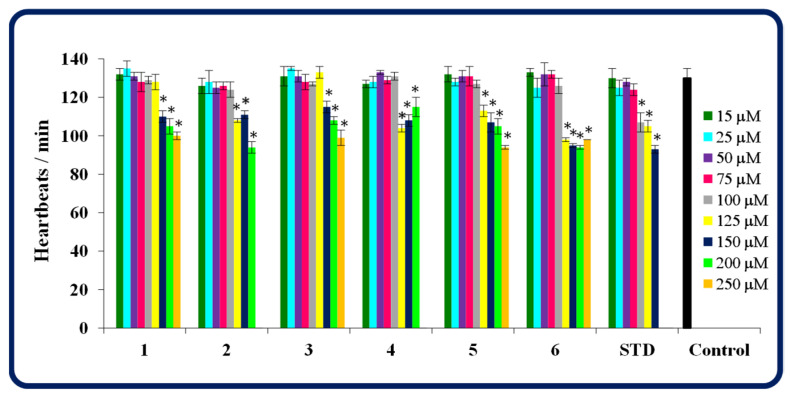
Cardiac function measured by heartbeats per minute in zebrafish exposed to the tested compounds/standard drug. STD—a standard drug pemetrexed. Data represent the mean ± SD of three independent experiments under similar conditions. *—statistically different from the control group (*p* < 0.05, Student’s *t*-test).

One of the first organs to develop in zebrafish is the heart. Due to its structural similarity to the human heart and the ease of observation owing to the transparency of the embryonic/larval body, the heart of *Danio rerio* is a very useful model to reveal the cardiotoxic effects of pharmacologically active compounds in the early phase of drug development. One of the factors that define cardiotoxicity is heart rate. The measurement of heartbeat is an important parameter in both embryonic development and the assessment of cardiac function because changes in heart rate may indicate developmental disorders or be both a cause and a consequence of pathological heart conditions [10,11,13,14]. In this study, to assess the cardiac function of compound/standard drug-treated and control zebrafish larvae, the heart rate per minute was calculated in each group. As shown in Figure 4, at the end of the exposure period (96 hpf), there were no significant effects of compounds **1** and **3** at concentrations up to 125 µM and molecules **2**, **4**, **5,** and **6** up to 100 µM on heart rate compared to the control. However, zebrafish treated with dihydroimidazotriazinones **1**–**6** at higher concentrations presented reduced heart rates. In turn, compared to the control group, no significant differences in the number of heartbeats were recorded in zebrafish exposed to pemetrexed only at concentrations up to 75 µM, while higher concentrations of this standard drug resulted in a remarkable decrease in heart rate.

**Figure 5:**
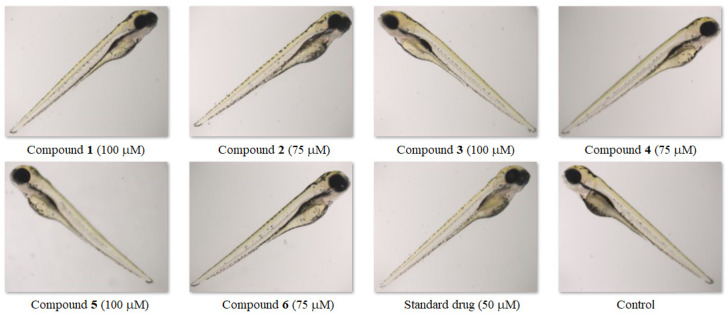
The representative 96 h old larvae from the control group and groups exposed to the highest concentration of compound/standard drug that did not induce phenotypic abnormalities.

**Figure 6:**
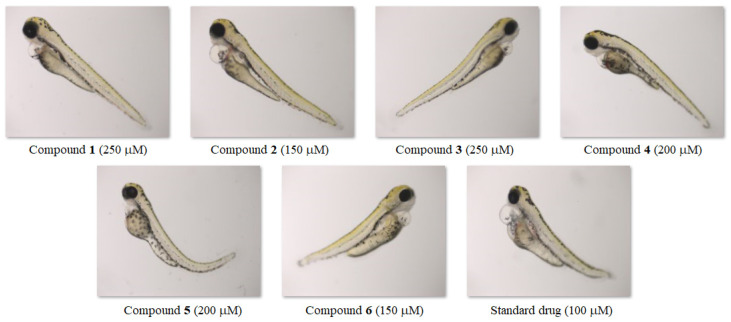
Phenotypic abnormalities (yolk sac swelling, pericardial oedema, and abnormal body shape) observed in 96 h old larvae treated with compounds **1**–**6** and the standard drug.

An important parameter for assessing the toxicity of pharmaceutical substances to zebrafish is whether these compounds induce any morphological abnormalities in their development [10,15]. This study showed that exposure to most dihydroimidazotriazinones tested had no significant effect on the developing zebrafish. The majority of larvae developed normally. Figure 5 presents the representative zebrafish larvae exposed to the highest concentration of compound/standard drug at which no adverse effects were observed. However, developmental defects, such as yolk sac/pericardium swelling and abnormal body shape, were found in zebrafish exposed to tested molecules only at the highest concentrations. It is noteworthy that these malformations were observed less frequently than in larvae exposed to the standard drug. The most severe developmental abnormalities in the zebrafish groups tested are shown in Figure 6.

Based on all the observed changes, NOAEC and LOAEC values were established for each of the tested compounds (Table 2). NOAECs for heterocycles **1**, **3,** and **5** amounted to 100 µM and for molecules **2**, **4,** and **6**—75 µM, while LOAECs were 75 and 50 µM, respectively. Interestingly, these values for all the investigated dihydroimidazotriazinones were found to be higher than those for the standard drug (50 and 75 µM, respectively), which proves they are safer for zebrafish than pemetrexed.

### 2.3. Assessment of the Effect on Red Blood Cells of the Tested Compounds (***1**–**6***)

Evaluation of the haemolytic potential of pharmacologically important compounds is a necessary part of the assessment of their toxicity. Mammal non-nucleated red blood cells represent a good model to study this cytotoxicity. Therefore, we first performed the ex vivo haemolysis assay to determine possible interactions of dihydroimidazotriazinones **1**–**6** with blood components as a necessary part of their biocompatibility in the preclinical phase of drug development. When examining their impact on erythrocytes, we found out that none of the tested compounds at 0.15 mM (an anticancer effective concentration) was able to promote any significant haemolytic effects. We showed that the haemolytic activity of molecules **1**–**6** was less than 7% compared to triton X-100 (a positive control causing total haemolysis), thus giving proof that the tested heterocycles are safe for red blood cells (Table 3). Haemocompatibility testing is needed for the development of safe drug candidates.

Next, we assessed the ability of dihydroimidazotriazinones **1**–**6** to inhibit oxidative haemolysis. For this purpose, red blood cells were pre-treated with each investigated heterocycle and then exposed to reactive oxygen species, i.e., AAPH-derived peroxyl radicals. Among the tested molecules, three compounds bearing 4-OCH_3_Ph (**3**), 4-CH_3_Ph (**2**), or Ph (**1**) showed the highest ability to protect erythrocytes from AAPH-induced haemolysis. The antihaemolytic activity of these molecules was found to be 85%, 72%, and 70% of the activity of ascorbic acid and 81%, 68%, and 67% of the activity of trolox (Table 3). These results indicate that dihydroimidazotriazinones **1**–**3** are able to effectively protect red blood cells from oxidative stress-induced damage.

### 2.4. Assessment of the Risk of Side Effects of the Studied Compounds (***1**–**6***)

Given the relatively clean toxicity profile and evidence of antiproliferative effects in some severe cancers and acute promyelocytic leukaemia, we decided to assess the in silico risk of adverse effects at the end of our biological studies. This risk may be a major problem in the case of potential pharmaceuticals; therefore, it has to be assessed in the preclinical phase of drug development. Mutagenic, tumorigenic, reprotoxic, and irritating effective molecules cannot be subjected to in vivo studies on animals to avoid unethical testing. To predict the possibility of the appearance of mutagenicity, tumorigenicity, irritating effects, and reproductive toxicity in the investigated class of dihydroimidazotriazinones (**1**–**6**), we applied the risk predictor—OSIRIS Property Explorer [17]. This useful in silico tool is able to locate each structural fragment, which gives rise to toxicity alerts, if it is present in the molecule studied. The result of predicting the risk score for each above-mentioned adverse effect is colour-coded. Green colour means no risk (score 1.0), yellow colour means medium risk (score 0.8), and red colour means high risk (score 0.6). Taking into account the in silico results, all the investigated compounds (**1**–**6**) were predicted to be non-mutagenic, non-carcinogenic, non-irritating effective, and non-reprotoxic (Table 4). This was as expected due to the lack of structural fragments in their molecules that would cause toxicity alerts. Such sets of heterocycles can be subjected to further in vivo studies. In turn, gemcitabine was predicted to be non-mutagenic, non-irritating effective, and non-reprotoxic but, unfortunately, tumorigenic, which was also observed in its in vitro studies [18,19].

### 2.5. Predicting Molecular Targets for the Investigated Compounds (***1**–**6***)

In order to assess the possible molecular targets for dihydroimidazotriazinones **1**–**6**, the Molinspiration Cheminformatics free web server [20] was used. Results of this in silico screening are presented as bioactivity scores (Table 5). The higher the value of the bioactivity score, the higher the probability of the molecule to be active. All the compounds mimic biogenic bicyclic nucleobases, and they were in silico screened as potential ligands modulating G-protein coupled receptors (GPCRs) and ion channels; enzyme, protease, and kinase inhibitors; and nuclear receptor ligands. According to the interpretation of bioactivity scores listed in Table 5, the investigated molecules should be moderately active as enzyme inhibitors and ligands modulating GPCRs. This may be due to the structural similarity of these modified bicyclic aza-analogues of isocytosine to biogenic bicyclic nucleobases, and their incorporation as fraudulent building blocks may lead to lethal synthesis by inhibiting enzymes participating in biochemical pathways [2]. In addition, this may probably be due to their similarity to some antagonists showing affinity for the two adenosine receptor subtypes (e.g., A_2A_ and A_2B_), belonging to the ligands that modulate GPCRs [21].

### 2.6. Evaluation of the Melting Parameters by the DSC Method

**Figure 7:**
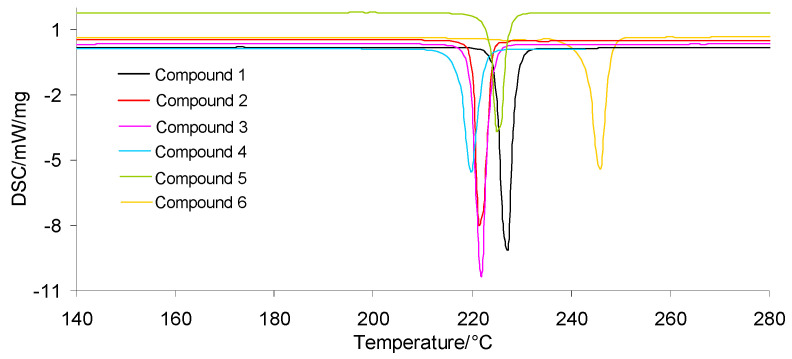
DSC curves for the tested compounds collected in an inert atmosphere.

The course of the melting process (the solid-liquid phase transition) of the tested dihydroimidazotriazinones is presented as the DSC curves in Figure 7. In addition, the melting parameters obtained in both furnace atmospheres (inert and oxidising) are placed in Table 6. Based on these results, it is concluded that all the analysed compounds melt within one narrow temperature range. This confirms that these are high-purity heterocycles. Furthermore, their transition from solid to liquid state (documented by one sharp endothermic peak in the DSC curve) occurred without thermal decomposition. It can be seen that the parent structure **1** and its derivatives **2**–**6** are molecules with a high melting point. Their melting temperatures depend on their structure and, more specifically, on the unsubstituted/substituted phenyl moiety, but in general, it can be said that their melting begins at temperatures above 216 and 217 °C in both used atmospheres. The highest melting temperature (above 243 °C) for compound **6** containing two chloro groups in *meta* and *para* positions of the phenyl moiety is confirmed. On the other hand, the lowest melting temperature (above 216 °C) is registered for heterocycle **4** bearing the *meta*-chlorophenyl substitution. If we look at the melting point values, we can arrange the analysed molecules as follows, starting from the highest melting point: compound **6** (R = 3,4-Cl_2_Ph) > compound **1** (R = Ph) = compound **5** (R = 4-ClPh) > compound **3** (R = 4-OCH_3_Ph) = compound **2** (R = 4-CH_3_Ph) > compound **4** (R = 3-ClPh). As can be seen from the attached results, the melting enthalpy (Δ*H*) values also depend on the structure of tested molecules. Compounds that do not contain the chlorine atom/atoms at the phenyl moiety have the highest Δ*H* values. The melting enthalpies for heterocycles **1**–**3** are within the range of 163.6–187.5 J/g. In contrast, derivatives containing at least one chlorophenyl substituent melt with Δ*H* values in the range of 116.3–140.9 J/g. These differences in enthalpy values between compounds with or without a chlorophenyl substituent indicate that less energy must be supplied to melt molecules with one (**4** and **5**) or two (**6**) chlorophenyl group/groups. This, in turn, suggests that the presence of chlorine atom/atoms at the phenyl moiety has a significant activating effect on the melting. Moreover, based on the values of melting point and melting enthalpy in both atmospheres (inert and oxidising), it can be concluded that the melting process occurs in a similar way, and the furnace atmosphere does not influence its course.

All dihydroimidazotriazinones subjected to polymorphism screening at low heating rates did not undergo any polymorphic transformations. This probably means that during the recrystallisation process from organic solvents, they formed a thermodynamically stable solid phase existing at the lowest free-energy level [4,7]. The advantage of these compounds in the crystalline form over amorphous substances is that, unlike the latter, they do not undergo polymorphic changes during long-term storage. This is an important finding because polymorphic forms differing in stability as well as in some physicochemical properties (e.g., melting point, density, solubility, and dissociation rate) can have a significant impact on the pharmacodynamics of pharmaceutical substances and, hence, on their clinical and toxicological effects [4,25].

### 2.7. Evaluation of the Thermal Properties by the TG/DTG Method (Inert Conditions)

**Figure 8:**
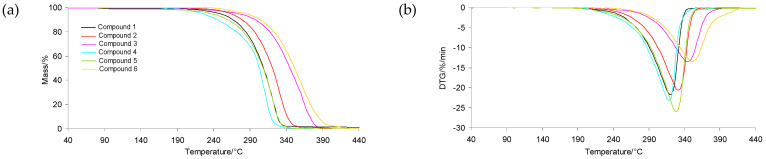
TG (**a**) and DTG (**b**) curves for the tested compounds in inert conditions.

The course of the thermogravimetry/differential thermogravimetry (TG/DTG) curves registered for the investigated compounds is presented in Figure 8. In turn, the values of temperatures characteristic of the decomposition of the tested heterocycles read from the TG/DTG curves are placed in Table 7. As it is well seen, all dihydroimidazotriazinones decompose in one main but wide stage in an inert atmosphere, which suggests that the pyrolysis process is related to several decomposition reactions with small differences in activation energies. Their decomposition up to a temperature of 450 °C is complete except parent structure **1**, for which a small residue (1.1%) is observed. In general, it can be said that the tested compounds are high thermally stable heterocycles. Their decomposition begins above a temperature of 230 °C in an inert furnace atmosphere. However, the initial decomposition temperatures, which describe the thermal stability of the investigated molecules, depend significantly on the type of substituent. If we look at heterocycles that do not contain the chlorophenyl moiety in their structure (compounds **1**–**3**), we can see that their thermal stability increases as follows: compound **1** (R = Ph) < compound **2** (R = 4-CH_3_Ph) < compound **3** (R = 4-OCH_3_Ph). Similarly, if we analyse the thermal stability results for structures with a chlorophenyl substitution, we can see that their thermostability increases as follows: compound **4** (R = 3-ClPh) < compound **5** (R = 4-ClPh) < compound **6** (R = 3,4-Cl_2_Ph). In addition, it can be noticed that molecules **4** and **5** with only one chlorine atom at the phenyl ring are characterised by the lowest thermal stability among heterocycles from this class (Table 7). In contrast, compound **6** bearing two chloro groups at this moiety is the most thermally stable of all the molecules studied. This indicates the stabilising effect of the two chlorine atoms at the phenyl ring and thus increasing the activation energy of the decomposition of compound **6**.

### 2.8. The Decomposition Course of the Tested Compounds (Inert Conditions)

**Figure 9:**
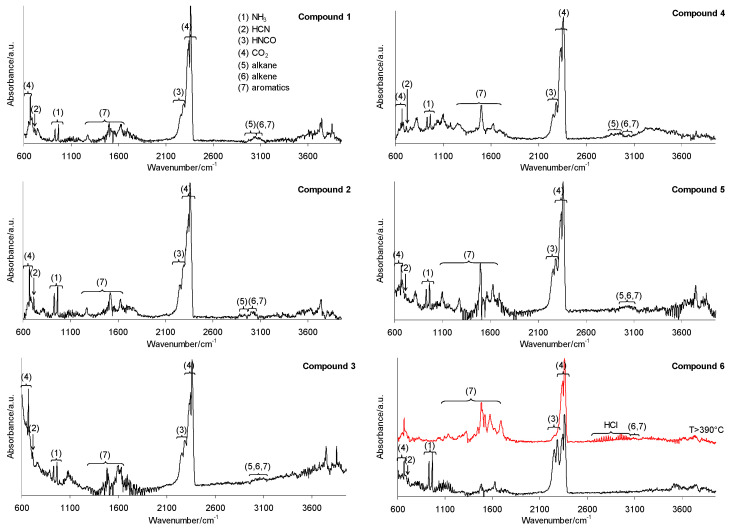
The gaseous FTIR spectra collected at *T*_max1_ in inert conditions.

**Figure 10:**
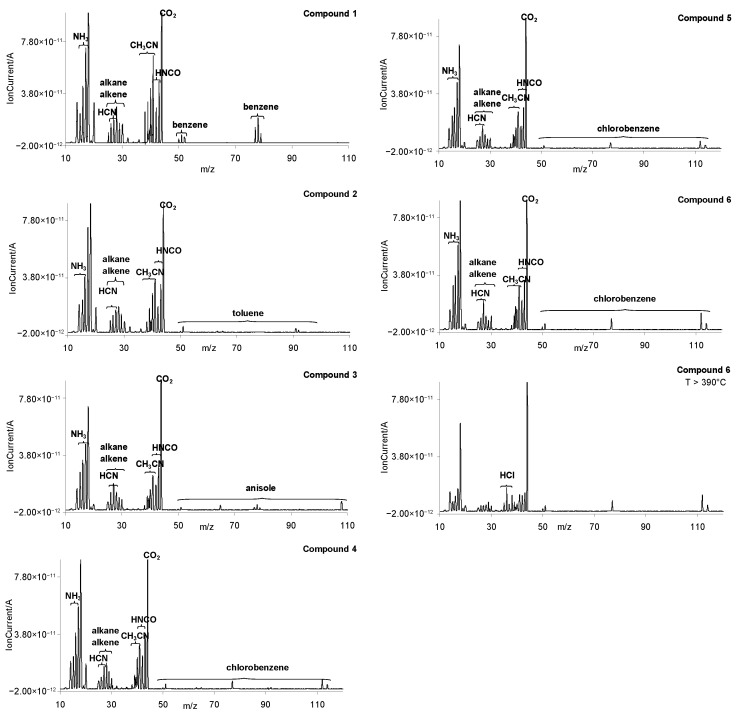
The gaseous QMS spectra collected at *T*_max1_ in inert conditions.

The decomposition course of dihydroimidazotriazinones **1**–**6** was assessed based on the type of gaseous decomposition products emitted. The released volatiles were characterised on the basis of FTIR and QMS spectra recorded by online analysers connected to the TG furnace. The gaseous FTIR spectra extracted at *T*_max1_ are presented in Figure 9. In turn, Figure 10 shows the gaseous QMS spectra gathered at *T*_max1_. Comparing the FTIR spectra of gaseous decomposition products for all tested compounds, it can be seen that the same type of volatiles at *T*_max1_ are emitted. The formation of NH_3_ is proved by the presence of two characteristic absorption bands, one at 931 cm^−1^ and another at 966 cm^−1^ in the FTIR spectra. These bands are due to the deformation vibrations of N–H groups [26,27,28,29]. Moreover, due to the ionisation of NH_3_, the *m*/*z* ions 15 (NH^+^), 16 (NH_2_^+^), and 17 (NH_3_^+^) in the QMS spectra further prove the emission of ammonia. The release of HCN is confirmed by the presence of one characteristic absorption band at 713 cm^−1^ and the *m*/*z* ions 26 (CN^+^) and 27 (HCN^+^) [30,31,32,33]. In addition, the QMS spectra prove the creation of CH_3_CN by the attendance of the *m*/*z* ions 41 (CH_3_CN^+^), 40 (CH_2_CN^+^), 39 (CHCN^+^), and 38 (CCN^+^) [34,35,36,37,38]. The absorption bands at 2270–2290 cm^−1^ indicate the creation of HNCO. Its presence is confirmed by the occurrence of the *m*/*z* ions 42 (NCO^+^) and 43 (HNCO^+^) in the QMS spectra. Additionally, the formation of CO_2_ is well visible for both FTIR and QMS spectra [39,40]. Besides these volatiles, the emission of alkane, alkene, and aromatic fragments by the attendance of the following absorption bands at 2880–2920 cm^−1^ (alkane), 3025–3027 cm^−1^ (alkene), 700–900 cm^−1^ (alkene, aromatics), 3050–3100 cm^−1^ (aromatics), and 1270–1600 cm^−1^ (aromatics) is confirmed. The creation of alkane and alkene fragments by the occurrence of the *m*/*z* ions in the range of 25–30, as marked in Figure 10, is proved. The most expected alkane and alkene fragments are ethane and ethylene and its polymerisation products. However, the type of emitted aromatic fragments clearly depended on the initial structure of heterocycles tested. When we look closely at the gaseous FTIR spectrum for parent compound **1**, we can see the following absorption bands at 673–720 cm^−1^ (out-of-plane deformation vibrations for C_ArH_), 1492–1600 cm^−1^ (stretching vibrations for C_Ar_=C_Ar_), and 3047–3080 cm^−1^ (stretching vibrations for C_ArH_). The presence of these bands indicates the formation of benzene as an aromatic gaseous decomposition product when compound **1** is heated (Figure 9).

The collected QMS spectra for all the tested molecules allow us to more precisely specify the type of aromatic products released during their decomposition. In Figure 10, the *m*/*z* ions characteristic for the ionisation of benzene are clearly visible. The presence of the *m*/*z* ions 77 (C_6_H_5_^+^), 78 (C_6_H_6_^+^), 79 (C_6_H_7_^+^), 50 (C_4_H_2_^+^), 51 (C_4_H_3_^+^), and 52 (C_4_H_4_^+^) directly confirms the creation of benzene upon heating of the parent structure **1**. In the case of compound **2**, the gaseous FTIR spectrum shows the attendance of the absorption bands typical for the creation of toluene. These are the stretching vibrations for C_ArH_ at 3043–3074 cm^−1^, the stretching vibrations for C-H at 2882–2935 cm^−1^, the stretching vibrations for C_Ar_=C_Ar_ at 1506–1607 cm^−1^, and the out-of-plane deformation vibrations for C_ArH_ at 694–728 cm^−1^. The formation of toluene as a decomposition product of molecule **2** is also proved by the presence of the *m*/*z* ions 91 (C_7_H_7_^+^), 92 (C_7_H_8_^+^), and 51 (C_4_H_3_^+^) in the QMS spectrum [41,42]. In turn, as the main aromatic decomposition product for heterocycle **3**, anisole is created. It is verified based on the absorption bands at 3000–3078 cm^−1^, 1502–1600 cm^−1^, 1178–1294 cm^−1^, 690–1048 cm^−1^, and the *m*/*z* ions 108 (C_7_H_8_O^+^), 78 (C_6_H_6_^+^), and 65 (C_5_H_5_^+^) [43]. In turn, the emission of chlorobenzene for the decomposition of dihydroimidazotriazinones **3**–**5** by the existence of the out-of-plane deformation vibrations at 736–780 cm^−1^, stretching vibrations for C-Cl at 810–815 cm^−1^, stretching vibrations for C_Ar_=C_Ar_ at 1480–1600 cm^−1^, and stretching vibrations for C_ArH_ at 3030–3080 cm^−1^ in the gaseous FTIR spectra is confirmed. The identification of the following *m*/*z* ions: 112 (C_6_H_5_^35^Cl^+^), 114 (C_6_H_5_^37^Cl^+^), and 77 (C_6_H_5_^+^) is proof of the chlorobenzene formation [44,45]. In addition, during the heating of compound **6**, the creation of HCl (the characteristic absorption bands in the range of 2600–3100 cm^−1^ and the *m*/*z* ions 35 (^35^Cl^+^), 36 (H^35^Cl^+^), 37 (^37^Cl^+^), and 38 (H^37^Cl^+^)) as a result of the separation of chlorine from the benzene ring is well visible above the temperature of 390 °C [46].

**Scheme 1:**
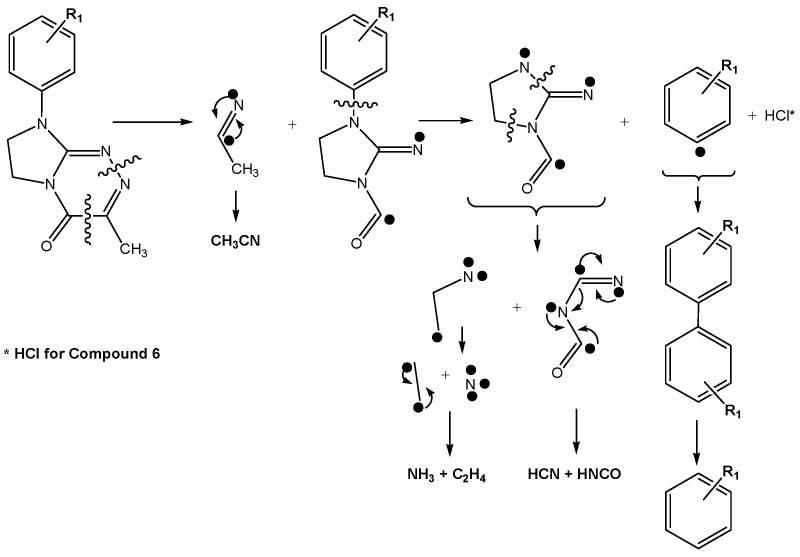
The course of pyrolysis of the tested compounds in inert conditions.

Based on these experimental observations, a diagram of the pyrolysis course of the tested compounds **1**–**6** was prepared and placed in Scheme 1.

### 2.9. Evaluation of the Thermal Properties by the TG/DTG Method (Oxidising Conditions)

**Figure 11:**
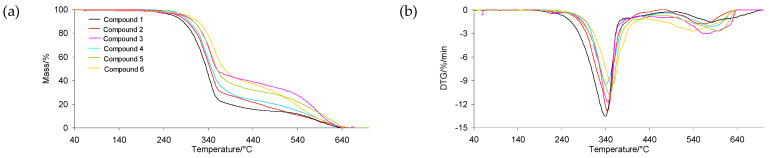
TG (**a**) and DTG (**b**) curves for the tested compounds in oxidising conditions.

Figure 11 shows the TG and DTG curves obtained for compounds **1**–**6** heated under oxidising conditions. The data read from TG/DTG curves are placed in Table 8. In the presence of air atmosphere, all dihydroimidazotriazinones decompose in two main stages. The first decomposition stage from *T*_5%_ to 430–500 °C with a similar maximum peak of decomposition (*T*_max1_) is observed. In this stage, the mass loss (Δ*m*_1_) is from 61.5% to 86.1%, and it is directly dependent on the structure of initial compounds. As a reminder, in inert conditions, based on the obtained TG/FTIR/QMS data, the investigated heterocycles decomposed completely up to a temperature of 450 °C. This indicated the pyrolysis processes leading to the simultaneous evaporation of released volatiles, both aromatic compounds and nitrogen ring decomposition products. However, as seen in Table 8, the mass loss in the first decomposition stage is lower up to a temperature of 450 °C, as compared to the mass loss in an inert atmosphere. This indicates a different mechanism of decomposition of the tested dihydroimidazotriazinones with the participation of oxygen leading to some oxidation reactions and/or combustion of the formed gaseous derivatives.

The thermal stability of molecules **1**–**6** is high in an oxidising atmosphere. It is from 261 °C (compound **1**) to 295 °C (compound **6**). This thermostability may prove useful in designing thermally stable drug candidates that can be stored at temperatures ranging from 20 to 40 °C without affecting their shelf life [47,48]. This means that dihydroimidazotriazinones **1**–**6** would be stable even in more adverse conditions in different climatic zones. Furthermore, high thermostability would be of importance in the case of approval of these analytes as pharmaceuticals. Based on the obtained thermal analysis results, it can be assumed that no special recommendations regarding the conditions of their storage will be required. It should be noted that in order to repay the research and development costs, an approved pharmaceutical substance must be acceptable worldwide. If the active pharmaceutical ingredient is exported, its stability must be predictable in a variety of climates.

The thermal stability of the investigated heterocycles increases with their increasing molar masses in the following order: compound **1** (R = Ph) < compound **2** (R = 4-CH_3_Ph) < compound **3** (R = 4-OCH_3_Ph) = compound **4** (R = 3-ClPh) < compound **5** (R = 4-ClPh) < compound **6** (R = 3,4-Cl_2_Ph). In addition, the thermal stability of molecules **1**–**3** and **6** is about 8–13 °C higher in the presence of oxygen than in an oxygen-free environment. The highest difference in thermal stability between the two furnace atmospheres is observed for compounds **4** and **5** containing one chlorine atom at the phenyl moiety (51.3 °C and 51.6 °C, respectively). These higher values prove higher activation energies of the decomposition process in oxidising conditions, which indicates that oxygen is an inhibitor of the decomposition reaction of the tested bicyclic polyazaheterocycles. The second decomposition stage is described by a DTG signal of low intensity but wide range with the *T*_max2_ from 543 to 603 °C. The mass loss (Δ*m*_2_) is in the range of 16.9–39.7%, and it is most likely related to the combustion reactions of the obtained residue in the first decomposition stage.

### 2.10. The Decomposition Course of the Tested Compounds (An Oxidising Atmosphere)

**Figure 12:**
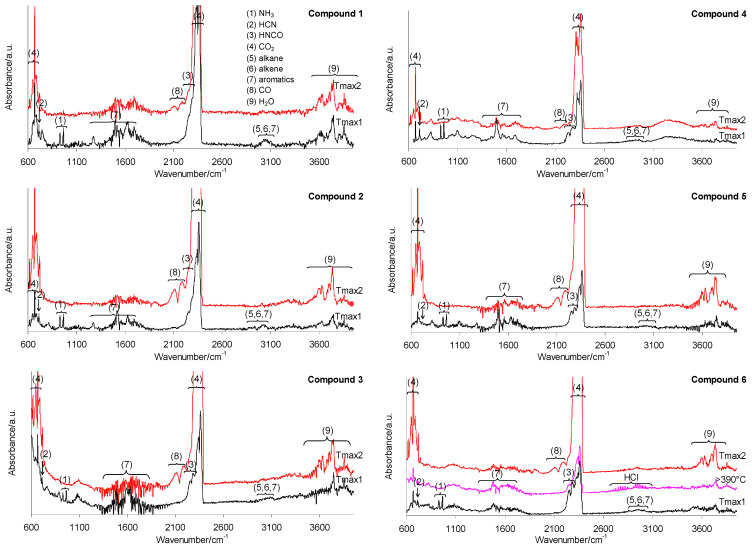
The gaseous FTIR spectra collected at *T*_max1_ and *T*_max2_.

**Figure 13:**
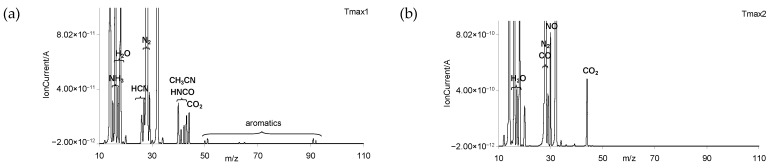
The exemplary QMS spectra collected at *T*_max1_ (**a**) and *T*_max2_ (**b**) (for compound **2**) in oxidising conditions.

The experimental gaseous FTIR spectra collected at *T*_max1_ and *T*_max2_ are presented in Figure 12. The exemplary QMS spectra gathered at *T*_max1_ and *T*_max2_ are also placed in Figure 13. As it is well seen, at *T*_max1_, the same gaseous decomposition products as those in an inert atmosphere are created. Among them should be mentioned: NH_3_, HCN, CH_3_CN, HNCO, alkane, alkene, and aromatic fragments, as marked in Figure 12 and Figure 13. However, a lower mass loss at the first decomposition stage in the presence of oxygen compared with this mass loss in inert conditions testifies about the formation of more stable derivatives with larger molecular masses and/or reactions with oxygen and, thus, these higher evaporation temperatures. From this, it follows that some of the decomposition products, the same as those observed in oxygen-free conditions, evaporate in the first stage, and some remain as a residue in the form of derivatives with higher molar masses, containing oxygen in their structures.

As the heating temperature increases to *T*_max2_, the higher emission of CO_2_ and H_2_O and the additional release of CO (FTIR: 2088–2167 cm^−1^, QMS: *m*/*z* ion 28 CO^+^) and NO (FTIR: 1858–1950 cm^−1^, QMS: *m*/*z* ion 30 NO^+^) prove the oxidation and combustion processes of previously formed residues. Moreover, the QMS spectra show the formation of N_2_ as a gaseous product (*m*/*z* ion 28 (N_2_^+^) and 14 (N^+^)). This molecule is invisible in the FTIR spectra due to its symmetrical structure. However, as Figure 13 shows, the presence of this gas is already detected at *T*_max1_ based on the QMS analysis. This confirms that oxidation reactions occur at this temperature.

Moreover, in the case of compound **6**, the emission of HCl above the temperature of 390 °C, just like in a non-oxidising atmosphere, is clearly visible.

## 3. Materials and Methods

### 3.1. Chemicals, Standard Drugs, and Kits

Pemetrexed, dimethyl sulfoxide (DMSO), tricaine (ethyl 3-aminobenzoate methanesulfonate, triton X-100 (polyethylene glycol *tert*-octylphenyl ether solution), 2,2′-azobis(2-amidinopropane)dihydrochloride (AAPH), ascorbic acid (AA), and trolox (6-hydroxy-2,5,7,8-tetramethylchroman-2-carboxylic acid) were purchased from Sigma-Aldrich (Saint Louis, MO, USA). 5-Bromo-2′-deoxyuridine Labeling/Detection Kit III was supplied from Roche Diagnostics GmbH (Indianapolis, IN, USA). Gemcitabine was obtained from Apollo Scientific Ltd. (Stockport, UK), and phosphate-buffered saline (PBS) was bought from Biomed (Lublin, Poland).

### 3.2. The Investigated Compounds (***1**–**6***)

The synthesis and structural assignment of the majority of investigated compounds (**2**–**6**) have previously been described [1]. Their structures in solution and solid state, as well as their purity, have been established with the use of spectroscopic methods (^1^H NMR and EI-MS) and TLC. Satisfactory analytical and spectral data of molecules **2**–**6** and their retention factor values have been reported [1]. All dihydroimidazotriazinones intended for biological and thermal studies were obtained from the appropriate 2-hydrazinylidene-1-(R-phenyl)imidazolidine and 2-oxopropanoic acid in one-pot cyclocondensation process. The pure samples of compounds **1**–**6** were obtained by recrystallisation of crude reaction products from DMF. 3-Methyl-8-phenyl-7,8-dihydroimidazo[2,1-*c*][1,2,4]triazin-4(6*H*)-one, which was required for the current research needs as the parent structure **1** was de novo resynthesised and, after recrystallisation from DMF, had the melting point of 225–226 °C, similar to the literature data (225–227 °C) [49]. Its ^1^H NMR spectrum in DMSO-d_6_ helped in identifying all the proton signals present within the molecule: the singlet at δ 2.25 ppm integrating with three protons of the methyl group (attached at *C*3), the singlet at δ 4.13 ppm integrating with four protons of ethylene moiety (present in the imidazolidine ring), and the multiplet at δ in the range of 7.09–7.86 ppm integrating with five methine protons belonging to the phenyl moiety. In this way, the parent compound **1** was identified based on its melting point, and its structure was confirmed in solution. All the investigated compounds (**1**–**6**) were stored in brown glass bottles at room temperature.

### 3.3. Assessment of the Anticancer Activity and Toxicity for Normal Cells of the Studied Compounds

All the investigated compounds (**1**–**6**) were assessed for their in vitro antiproliferative activity—presented as the percentage growth inhibition—against cancer cells derived from four solid tumours: A549 (ECACC 86012804)—human Caucasian lung carcinoma cells; HeLa (ECACC 93021013)—human Negroid cervix epitheloid carcinoma cells; TOV112D (ATCC CRL-11731)—human ovarian primary malignant adenocarcinoma cells; and T47D (ECACC 85102201)—human breast carcinoma cells. One malignant cell line derived from human acute promyelocytic leukaemia (HL-60) was also included in the anticancer screening. In addition, each molecule was evaluated for its cytotoxicity in non-tumour African green monkey kidney cells (GMK-ECACC 88020401). For this purpose, the BrdUrd bioassay was used, which is based on the quantitative measurement of DNA synthesis and allows for the assessment of the proliferative capacity of cells [50,51,52]. All the molecules were tested at a concentration of 0.15 mM after 24-, 48-, and 72-h incubation periods according to the procedure described previously [53]. For comparison purposes, the approved anticancer drug gemcitabine was used at the same concentration. To evaluate the antitumour activity and cytotoxicity to normal cells of the investigated compounds, the optical densities of all samples were measured using an ELISA reader (BIO-TEK Instruments Inc., Winooski, VT, USA).

### 3.4. Zebrafish Toxicity Studies

#### 3.4.1. Preparing Solutions of the Tested Compounds

All the tested compounds (**1**–**6**) and standard drug (pemetrexed) were dissolved in DMSO and diluted with an embryonic medium (E3 medium: a purified water with 17.4 µM NaCl, 0.21 µM KCl, 0.18 µM Ca(NO_3_)_2_, and 0.12 µM MgSO_4_, pH = 7.1–7.3) to achieve suitable concentrations ranging from 15 to 300 µM.

#### 3.4.2. Zebrafish Breeding and Egg Collection

Adult wild-type zebrafish (AB strain) were bred at the Experimental Medicine Centre of the Medical University of Lublin, Poland. Fish were maintained in a breeding room under a 14 h light: 10 h dark photoperiod cycle in a water-recirculating system at a temperature of 28 ± 0.5 °C. Zebrafish eggs were obtained by random mating between sexually mature individuals (males and females in a 2:1 ratio).

#### 3.4.3. Fish Embryo Acute Toxicity (FET) Test

The Fish Embryo Acute Toxicity (FET) test was carried out on zebrafish (*Danio rerio*) in accordance with Guidelines for the Testing of Chemicals (Guideline No. 236) recommended by the Organization for Economic Cooperation and Development (OECD) [16].

Briefly, a half hour post-fertilisation, eggs were transferred to Petri dishes (Costar, Corning Inc., Glendale, AZ, USA) (100 eggs/dish) filled with freshly prepared E3 medium. All collected eggs were visualised under the SteREO Discovery V8 optical microscope with a camera (Carl Zeiss Microscopy GmbH, Göttingen, Germany) to remove unfertilised, coagulated, and damaged eggs. Then, fertilised and well-developing eggs were distributed into sterile 24-well plates (Costar, Corning Inc., Glendale, AZ, USA), one embryo per well, filled with 2 mL of a solution of tested compounds. Embryos were exposed for 96 h to solutions of compounds **1**–**6** (treated groups) or pemetrexed (positive control groups) or to the E3 medium only (a negative control group). In each plate (representing one tested group), 20 embryos were exposed to compound/standard drug at one of the 10 concentrations tested (15, 25, 50, 75, 100, 125, 150, 200, 250, or 300 µM), while 4 embryos were exposed to E3 medium (a negative internal control). The concentration of DMSO used as a solvent in our studies had no effect on zebrafish development, so a DMSO control group was not necessary. On each day of exposure, the freshly prepared test solutions were replaced in each well to ensure the real concentrations of the test solutions did not fall under 80% of their nominal values. During the experiment, the covered plates were kept in an incubator (IN 110 Memmert GmbH, Schwabach, Germany) under controlled temperature (28 ± 0.5 °C) and photoperiod (12 h light/12 h dark). Mortality, hatching rate, heart rate, and the type of developmental defects were observed under a stereomicroscope at 24 h intervals up to 96 hpf. After completing the research, the larvae were euthanised by an overdose of tricaine (300 mg L^−1^ solution). All experiments were performed in triplicate under similar conditions.

The acute toxicity of test compounds and a standard drug was assessed based on lethal and sublethal endpoints. Considering the mortality of zebrafish, the maximal non-lethal concentration (MNLC, maximum concentration of compound that does not result in statistically different mortality of zebrafish embryos/larvae compared to the control group during the experimental period) and the half-maximal lethal concentration (LC_50_, lowest concentration of compound causing 50% mortality of zebrafish embryos/larvae during the experiment) were calculated (using the probit method [54]) for each tested compound and standard drug. Additionally, the hatchability (number of hatched larvae at 96 hpf) and heartbeat rate (number of beats per minute) were quantified as well as developmental abnormalities (e.g., reduced yolk sac resorption, oedema formation, and body deformities) were qualitatively evaluated in zebrafish exposed to tested compounds or pemetrexed. Based on all assessed endpoints, the highest concentration at which no adverse effects are observed (NOAEC, no-observed-adverse-effect-concentration) and the lowest concentration at which adverse effects are observed (LOAEC, lowest observed adverse effect concentration) were established for each compound and standard drug.

#### 3.4.4. Ethical Statement

All experimental procedures on zebrafish were conducted in accordance with applicable national and international law. According to European legislation on the protection of animals used for scientific purposes, ethical approval for this study was not required since zebrafish embryos and larvae below 120 h old are not protected animal stages [55].

### 3.5. An Impact of the Investigated Compounds on Erythrocytes

The evaluation of haemolytic and antihaemolytic activities of molecules **1**–**6** was performed ex vivo on a 4% suspension of red blood cells in phosphate-buffered saline (PBS, pH 7.4). Erythrocytes were obtained by centrifugation (1500 rpm; 10 min; 4 °C) of sheep blood (Biomaxima, Lublin, Poland). The haemolytic potential of compounds was assessed in the haemolytic assay, while their antihaemolytic activity was established in the AAPH-induced haemolysis inhibition assay, according to the procedures described earlier [56]. To evaluate both the haemolytic potential and antihaemolytic activity of the studied compounds, absorbances of the samples were recorded by using a Hitachi U2800 UV-Vis spectrophotometer (Tokyo, Japan).

### 3.6. Evaluation of the Melting Parameters Using the DSC Method

The DSC method was applied to determine the melting parameters, such as the onset melting temperatures (*T*_onset_), the maximum melting temperatures (*T*_melt_), and the melting enthalpies (Δ*H*). For this purpose, 10 mg of each tested compound in an aluminium crucible with a pierced lid from 20 °C to 280 °C was heated. The heating rate was 10 °C min^−1^. The DSC analyses in inert (helium gas with a flow rate of 40 mL min^−1^) and in oxidising (synthetic air with a flow rate of 100 mL min^−1^) atmospheres were carried out. As the measuring instrument, a DSC 204 Phoenix produced by the Netzsch (Selb, Germany) was used.

### 3.7. Evaluation of the Thermal Properties and the Decomposition Course with the Use of Simultaneous TG/DTG Method Coupled with FTIR/QMS

The TG/DTG method was applied to evaluate the thermal properties of the tested compounds, such as the initial decomposition temperature marked as a 5% mass loss (*T*_5%_), peak maximum decomposition temperature (*T*_max_), mass loss in each decomposition stage (Δ*m*), and residual mass (rm). For this purpose, a 10 mg sample of each tested compound in an open corundum crucible from 40 °C to 450 °C (an inert atmosphere) or 700 °C (an oxidising atmosphere) was heated. The heating rate was 10 °C min^−1^. The TG/DTG analyses in inert (helium gas with a flow rate of 40 mL min^−1^) and in oxidising (synthetic air with a flow rate of 100 mL min^−1^) atmospheres were performed. A STA 449 Jupiter F1 instrument produced by Netzsch (Selb, Germany) was used.

In order to evaluate the decomposition course, the simultaneous TG/DTG method coupled with FTIR/QMS analysers was used to define and analyse the type of gaseous products emitted during the thermal decomposition. During the TG/DTG analysis, spectra of the emitted volatiles using online FTIR (FTIR TGA 585, Bruker, Mannheim, Germany) and QMS (QMS 403 C Aëolos, Netzsch, Selb, Germany) analysers were gathered. The gaseous FTIR spectra in the wavenumber range of 600–4000 cm^−1^ and with a resolution of 4 cm^−1^ and 32 scans per spectrum were recorded. The gaseous QMS spectra in the range of 10–150 amu were collected.

## 4. Conclusions

Taking into account the comprehensive results of in vitro, in vivo, and ex vivo biological studies, the investigated dihydroimidazotriazinones may represent a promising class of heterocyclic molecules with therapeutic value. These heterocycles mimicking innovative bicyclic polyazanucleobases may have potential in the treatment of human solid tumours and acute promyelocytic leukaemia. Compounds **1**, **3**, **4**, **5,** and **6**—with more favourable selectivity than anticancer agent gemcitabine—should be subjected to more extended investigations to assess their potential usefulness in cancer chemotherapy. It is worth noting that all the tested molecules exhibit a favourable toxicity/safety profile in both animal and cellular models. Comparative studies with pemetrexed showed that heterocycles **1**–**6** are safer for the early-life stages of zebrafish than this clinically approved anticancer agent, as their MNLC, LC_50_, NOAEC, and LOAEC values were higher than those of the standard drug. In addition, less severe and less frequent developmental abnormalities were observed in compound-treated than pemetrexed-treated groups, indicating their lower toxicity. Furthermore, none of the studied heterocycles produced haemolysis, and molecules **1**, **2,** and **3** were able to effectively protect red blood cells from the oxidative stress-induced damage. As a result, the vast majority of dihydroimidazotriazinones proved to be active and selective in preclinical studies; therefore, further in vivo investigations on this class of potential drug candidates are needed.

Considering the results of thermal studies, the article provides insight into the first detailed analysis of the thermal decomposition mechanism and pathway for this series of dihydroimidazotriazinones, which is of scientific value. The DSC analyses confirmed high purity of the tested compounds, their high melting points, and the dependence of the melting points and melting enthalpies on the presence of the substituted/unsubstituted phenyl moiety in their structure. In turn, the TG/DTG analyses proved high thermal stability of all the investigated heterocycles. The thermal stability of particular molecules depended directly on the type of substitution. Their pyrolysis process occurred in one main stage connected with the emission of NH_3_, HCN, HNCO, CH_3_CN, CO_2_, alkene, alkane, aromatics (in case of all the compounds), and HCl (in case of a molecule bearing the 3,4-Cl_2_Ph). The type of pyrolysis volatiles indicated the homolytic cleavage of C–C, C–N, and N–N bonds and the formation of radicals capable of further chemical reactions. In turn, the oxidative decomposition of compounds ran through at least two main stages and resulted in the release of the same gases as during pyrolysis in inert conditions and additional emissions of CO, H_2_O, NO, and N_2_. It was proven that this process proceeded through the radical mechanism combined with the reactions with oxygen and the combustion reactions of the formed intermediate products. In conclusion, high thermal stability and high purity of each compound as well as the absence of polymorphic transformations in the solid state seem to enhance the utility of this class of dihydroimidazotriazinones and incline them for future pharmaceutical applications.

This interdisciplinary research led to the documentation of favourable anticancer activity, toxicity profile, and thermal properties of a number of the original polyazaheterocycles. Therefore, these molecules appear to be suitable for future development in the drug research process.

## Figures and Tables

**Table 1:** Antiproliferative activities and cytotoxicities of the investigated compounds (**1**–**6**) in tumour and non-tumour cell lines.

Comp.	R	Incubation Time	Antiproliferative Activity					
			GMK	A549	HeLa	TOV112D	T47D	HL-60
**1**	Ph	24 h	0	10 ± 0.1	50 ± 0.4 **	50 ± 0.3 *	10 ± 0.2	0
		48 h	10 ± 0.1	50 ± 0.3 **	80 ± 0.4 **	80 ± 1.2 **	20 ± 0.2 *	15 ± 0.2
		72 h	30 ± 0.2 *	90 ± 0.7 **	100 ± 1.2 **	98 ± 0.9 **	90 ± 1.6 **	25 ± 0.5 *
**2**	4-CH_3_Ph	24 h	0	15 ± 0.5	50 ± 0.3 *	60 ± 1.2 **	10 ± 0.1	5 ± 0.3
		48 h	15 ± 0.1	60 ± 0.3 *	60 ± 0.4 *	90 ± 0.9 **	25 ± 0.3 *	25 ± 0.8 *
		72 h	75 ± 0.5 **	90 ± 0.8 **	100 ± 0.9 **	100 ± 1.5 **	95 ± 1.2 **	45 ± 0.7 *
**3**	4-OCH_3_Ph	24 h	0	20 ± 0.1	90 ± 0.8 **	40 ± 0.6 **	10 ± 0.2	15 ± 0.6
		48 h	20 ± 0.2 *	60 ± 0.4 **	98 ± 0.8 **	85 ± 0.9 **	15 ± 0.2	20 ± 0.6
		72 h	40 ± 0.3 *	90 ± 0.6 **	100 ± 0.9 **	100 ± 1.5 **	100 ± 0.9 **	45 ± 1.5 *
**4**	3-ClPh	24 h	0	10 ± 0.2	80 ± 0.5 **	55 ± 1.3 *	10 ± 0.5	20 ± 0.7
		48 h	25 ± 0.2 *	75 ± 0.5 **	100 ± 0.7 **	90 ± 0.7 **	45 ± 0.8 **	25 ± 0.8 *
		72 h	35 ± 0.4 *	85 ± 0.5 **	100 ± 0.9 **	100 ± 0.9 **	85 ± 1.2 **	50 ± 1.4 *
**5**	4-ClPh	24 h	0	15 ± 0.2	90 ± 0.7 **	35 ± 1.4 *	5 ± 0.2	10 ± 0.5
		48 h	35 ± 0.2 *	65 ± 0.4 *	100 ± 1.1 **	95 ± 0.8 **	15 ± 0.4	15 ± 0.6
		72 h	40 ± 0.3 *	90 ± 0.6 **	100 ± 0.9 **	100 ± 0.8 **	75 ± 0.7 **	55 ± 1.6 *
**6**	3,4-Cl_2_Ph	24 h	0	20 ± 0.2	75 ± 1.2 **	30 ± 0.8 *	0	25 ± 0.8
		48 h	15 ± 0.1	75 ± 0.6 **	100 ± 0.9 **	90 ± 1.8 **	75 ± 1.6 **	35 ± 1.5 *
		72 h	30 ± 0.2 *	95 ± 0.7 **	100 ± 0.9 **	100 ± 1.6 **	100 ± 1.4 **	45 ± 1.8 *
Standard drug		24 h	0	25 ± 0.7	25 ± 0.8 *	35 ± 0.7 *	2 ± 0.1	15 ± 0.5
		48 h	45 ± 1.3 *	80 ± 1.6 **	80 ± 1.2 **	75 ± 0.9 **	5 ± 0.3	25 ± 1.4 *
		72 h	55 ± 0.9 *	95 ± 1.8 **	100 ± 0.9 **	100 ± 1.8 **	55 ± 0.8 **	35 ± 0.9 *

A non-tumour cell line: GMK (ECACC 88020401)—African green monkey kidney cells. Tumour-derived cell lines: A549 (ECACC 86012804)—human Caucasian lung carcinoma cells; HeLa (ECACC 93021013)—human Negroid cervix epitheloid carcinoma cells; TOV112D (ATCC CRL-11731)—human ovarian primary malignant adenocarcinoma cells; T47D (ECACC 85102201)—human breast carcinoma cells. Leukaemia-derived cell line: HL-60—human acute promyelocytic leukaemia cells. Compounds and a standard drug gemcitabine were tested at a concentration of 0.15 mM. Data (from five independent experiments) are provided as the mean ± standard deviation. * *p* < 0.05; ** *p* < 0.001.

**Table 2:** The maximal non-lethal concentration (MNLC), the half-maximal lethal concentration (LC_50_), the no-observed-adverse-effect-concentration (NOAEC), and the lowest observed adverse effect concentration (LOAEC) values of the tested compounds (**1**–**6**) and standard drug for zebrafish.

Compound	R	MNLC (µM) ^a^	LC50 (95% CL ^b^, µM)	NOAEC (µM)	LOAEC (µM)
**1**	Ph	100 ± 0.0	149 (125–178)	100	125
**2**	4-CH_3_Ph	75 ± 0.0	115 (94–141)	75	100
**3**	4-OCH_3_Ph	117 ± 14.4	153 (128–183)	100	125
**4**	3-ClPh	92 ± 14.4	140 (119–166)	75	100
**5**	4-ClPh	100 ± 0.0	144 (119–175)	100	125
**6**	3,4-Cl_2_Ph	83 ± 14.4	119 (93–153)	75	100
Standard drug ^c^		58 ± 14.4	104 (85–127)	50	75

^a^ The mean ± SD of three independent experiments under similar conditions. ^b^ CL—confidence limit. ^c^—pemetrexed.

**Table 3:** Haemolytic and antihaemolytic activity of the investigated compounds (**1**–**6**).

Compound	R	Haemolytic Activity (%) in Relation to Triton X-100	Antihaemolytic Activity (%) in Relation to	
			AA	Trolox
**1**	Ph	6.99 ± 0.39	70 ± 6.1	67 ± 4.8
**2**	4-CH_3_Ph	5.42 ± 0.29	72 ± 5.8	68 ± 5.1
**3**	4-OCH_3_Ph	3.93 ± 0.22	85 ± 6.0	81 ± 7.1
**4**	3-ClPh	4.13 ± 0.37	59 ± 4.1	56 ± 3.3
**5**	4-ClPh	5.08 ± 0.30	64 ± 4.8	63 ± 4.2
**6**	3,4-Cl_2_Ph	5.35 ± 0.51	68 ± 5.4	64 ± 3.7

Triton X-100—polyethylene glycol *tert*-octylphenyl ether solution. AA—ascorbic acid. Trolox—6-hydroxy-2,5,7,8-tetramethylchroman-2-carboxylic acid. Compounds **1**–**6**, AA, and trolox were tested at a concentration of 0.15 mM. Data (from three independent experiments) are shown as the mean ± standard deviation.

**Table 4:** Risk assessment of adverse side effects for the studied compounds (**1**–**6**).

Compound	Drug Score	Mutagenicity	Tumorigenicity	Irritating Effects	Reproductive Toxicity
**1**	0.89	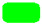	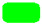	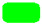	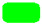
**2**	0.86	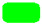	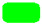	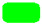	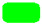
**3**	0.88	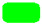	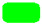	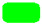	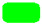
**4**	0.82	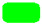	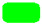	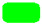	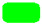
**5**	0.82	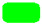	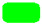	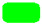	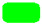
**6**	0.71	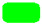	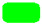	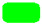	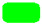
GEM	0.29	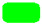	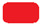	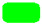	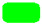

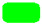
—no risk, score: 1.0. 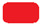
—high risk, score: 0.6. GEM—gemcitabine hydrochloride.

**Table 5:** Prediction of the bioactivity score for various molecular targets.

Compound	Bioactivity Score					
	GPCR Ligand	Ion Channel Modulator	Enzyme Inhibitor	Protease Inhibitor	Kinase Inhibitor	Nuclear Receptor Ligand
**1**	−0.72	−0.97	−0.63	−1.34	−0.92	−1.42
**2**	−0.67	−0.98	−0.62	−1.26	−0.87	−1.30
**3**	−0.60	−0.95	−0.57	−1.17	−0.77	−1.17
**4**	−0.64	−0.92	−0.64	−1.31	−0.83	−1.32
**5**	−0.63	−0.92	−0.61	−1.27	−0.84	−1.30
**6**	−0.55	−0.86	−0.58	−1.19	−0.73	−1.18

GPCR—G-protein-coupled receptor. The bioactivity score interpretation for organic molecules: above 0.00—active; in the range from −5.00 to 0.00—moderately active; below −5.00—inactive [22,23,24].

**Table 6:** DSC results for the tested compounds.

Compound	R	Inert Atmosphere			Oxidative Atmosphere		
		*T*_onset_/°C	*T*_melt_/°C	Δ*H*/J/g	*T*_onset_/°C	*T*_melt_/°C	Δ*H*/J/g
**1**	Ph	224.7	226.8	172.8	223.5	225.3	175.3
**2**	4-CH_3_Ph	219.6	221.3	163.6	218.6	220.4	164.6
**3**	4-OCH_3_Ph	219.6	221.7	185.5	218.3	220.7	187.5
**4**	3-ClPh	217.5	219.6	139.2	216.2	218.7	140.9
**5**	4-ClPh	223.6	225.3	116.3	222.1	224.6	118.4
**6**	3,4-Cl_2_Ph	243.3	245.7	135.5	242.2	244.3	137.2

*T*_onset_—onset melting temperature. *T*_melt_—maximum melting temperature. Δ*H*—melting enthalpy.

**Table 7:** TG/DTG data for the tested compounds in inert conditions.

Compound	R	Decomposition Process			
		First Decomposition Stage			
		*T*_5%_/°C	*T*_max1_/°C	Δ*m*_1_/%	rm/%
**1**	Ph	248.6	320.5	98.9	1.1
**2**	4-CH_3_Ph	263.3	331.6	100	0
**3**	4-OCH_3_Ph	274.7	344.6	100	0
**4**	3-ClPh	230.2	321.3	100	0
**5**	4-ClPh	236.7	328.6	100	0
**6**	3,4-Cl_2_Ph	284.2	351.2	100	0

*T*_5%_—initial decomposition temperature marked as a 5% mass loss. *T*_max1_—peak maximum decomposition temperature. Δ*m*_1_—mass loss. rm—residual mass at 450 °C.

**Table 8:** TG/DTG data collected for the tested compounds in oxidising conditions.

Comp.	R	Decomposition Process				
		First Decomposition Stage			Second Decomposition Stage	
		*T*_5%_/°C	*T*_max1_/*T*_max1′_/°C	Δ*m*_1_/%	*T*_max2_/°C	Δ*m*_2_/%
**1**	Ph	261.4	341.3	86.1	583.3	16.9
**2**	4-CH_3_Ph	272.4	344.0	82.9	560.3	17.1
**3**	4-OCH_3_Ph	282.2	345.1	65.7	571.0	35.3
**4**	3-ClPh	281.5	338.3	79.2	585.3	20.8
**5**	4-ClPh	288.3	349.1	69.6	603.1	27.1
**6**	3,4-Cl_2_Ph	295.5	355.8	61.5	543.5	39.7

*T*_5%_—initial decomposition temperature marked as a 5% mass loss. *T*_max1_. *T*_max1′_. *T*_max2_—peak maximum decomposition temperature in the first or second decomposition stage. Δ*m*_1_. Δ*m*_2_—mass loss in the first or second decomposition stage.

## Data Availability

The data presented in this study are available on request from the authors.
